# Experimental model in porcines to evaluate solutions used in endoscopic resections

**DOI:** 10.1016/j.igie.2024.06.004

**Published:** 2024-07-02

**Authors:** Annita Cavalcante Farias Leoncio, Carlos Kiyoshi Furuya, Christiano Makoto Sakai, Paulo Sakai, Edson Ide

**Affiliations:** Department of Gastrointestinal Endoscopy Unit, Oswaldo Cruz German Hospital, São Paulo, Brazil

## Abstract

**Background and Aims:**

Injection of solution into the submucosa is an essential step in endoscopic resections and aims to separate the mucosal layer from the muscular layer avoiding, above all, perforations. This study compares the durability of the solution in bubble formation, endoscopists' opinions on the quality of the bubble during resections, and the electrical resistance of the solutions.

**Methods:**

This double-blind study compared the following solutions: Blue Eye (B-bluee), Voluven (Voluv; 6% hydroxyethyl starch solution), 10% mannitol with .45% sodium chloride (Ma-Na45), and mannitol 20% with .9% sodium chloride (Ma-Na90). In Phase 1, a total of 5 mL of the solution was injected into the gastric antrum of a live pig; in Phase 2, two endoscopists performed 20 resections over 5 weeks; and during Phase 3, the study measured the electrical resistance of pure solutions and after injections into the submucosa of ex vivo gastric tissue.

**Results:**

Ma-Na90 lasted the longest (28 minutes), while Ma-Na45 had the shortest duration (10 minutes). Ma-Na45 was satisfactory, and Voluv was unsatisfactory. Ma-Na90 produced the most foam, and Voluv required the longest dissection time. There was no perforation. Ma-Na45 had the highest average electrical resistance (89.62 Ω) in both the solution and tissue (391 Ω), while Ma-Na90 exhibited lower values (23 Ω and 55.75 Ω, respectively).

**Conclusions:**

This experiment shows the level of complexity in choosing the best solution based on a combination of variables. Mannitol with sodium chloride stood out as a highlighted option due to its favorable overall results and easy accessibility.

EMR and endoscopic submucosal dissection (ESD) are minimally invasive and efficient techniques for removing premalignant lesions and early cancers from the GI tract. These techniques preserve organ functionality and have long-term outcomes comparable to those of surgery.^1^, ^2^, ^3^, ^4^

Injecting the solution into the submucosa is an essential step in these procedures. The aim is to elevate the lesion and separate it from the muscular layer, thereby reducing the risk of thermal injury, perforation, and bleeding, while simultaneously improving technical feasibility.^5^^,^^6^ Several types have been used for submucosal injection, although there is still no consensus on which option is the best.^7^

The ideal solution should create a durable fluid layer in the submucosal space, ensuring prolonged visibility, effective electrical conduction, absence of adverse events, and accessibility.^1^^,^^5^^,^^8^, ^9^, ^10^, ^11^, ^12^ In addition to not interacting with tissue samples, a good margin must be allowed for effective pathologic staging.^13^ The use of dyes in the composition is an additional factor, making the margins of the lesion clearer and more defined.^14^

An important and often overlooked aspect is the electrical conductivity of the submucosal injection fluids that would also influence the electrical circuit. In this context, recognizing the significance of measuring the electrical resistivity of solutions, which indicates the ease or difficulty of electric current passage, is essential for enhancing intervention outcomes.^15^

Among the injectable solutions, normal saline has been the most widely used. It is easy to use, readily available, and cost-effective, although the bubble created by submucosal injection of normal saline is only maintained for a short period. This solution can be used in various concentrations: .9%, 0%, 45%, or hypertonic 3%. Although this may not be an issue when removing small lesions, the need for repeated injections can increase procedure time in the resection of larger lesions and the risks of adverse events.^5^^,^^6^^,^^9^

To develop the ideal solution, several types of alternatives were tested based on glucose, glycerol, sodium hyaluronate, colloids, hydroxyethyl starch, hydroxypropyl methylcellulose, fibrinogen solution, and sodium alginate.^10^^,^^16^, ^17^, ^18^, ^19^ The process can be challenging, including difficulty with preparation, administration, high cost, and tissue interaction or even possible associations with toxicity.^1^^,^^5^, ^6^, ^7^, ^8^^,^^18^^,^^20^

The aim of the current experiment was to compare the durability of the solution during cushion formation, the subjective endoscopists’ opinion regarding the quality of the bubble during resections, and the electrical resistance of the solutions.

## Methods

This double-blind comparative experimental study was conducted in live and ex vivo porcine models at a surgical training center. The procedures were conducted on pigs weighing around 40 kg and using an ex vivo porcine stomach.

The study was divided into 3 phases. In the first phase, the maintenance time of a cushion was assessed by injecting solutions into the submucosa of the live porcine stomach. A total of 5 mL of the randomly chosen solution was injected into the antrum of the porcine using a 23-gauge sclerotherapy needle (Olympus, Tokyo, Japan). The cushion size was measured and compared with a 30 mm polypectomy snare. Measurements were recorded through photographs, from the initial appearance until it disappeared. All ESD procedures were performed with a GIF-H170 upper endoscope (Olympus). A VIO 300 D electrosurgical generator (Erbe Elektromedizin GmbH, Tübingen, Germany) with the ENDO CUT effect 3 electrosurgery settings was used.

In the second phase, 2 experienced endoscopists performed 20 ESDs over 5 weeks, conducting 4 procedures weekly. After each resection, they completed a form designed to assess the solution’s quality (rated as satisfactory, neutral, or unsatisfactory). The quality was organized and order of the performance ranked from 1 to 4, according to the endoscopists’ experience. The best experiences received a score of 4, those with 3 or 2 were considered neutral, and the last one received a score of 1 (unsatisfactory). The presence of foam formation, the solution's association with dissection time, and bubble dispersion were also evaluated to determine if there was any incidence of perforation.

Phases 2 and 3 were performed with injectable solutions prepared in identical syringes by a technician and randomly labeled as A, B, C, or D. The researcher and endoscopists remained blinded to the specific type of solution used. Experienced endoscopists rated the preferred order of solutions each week without knowing the real label right after the procedure.

**Figure 1:**
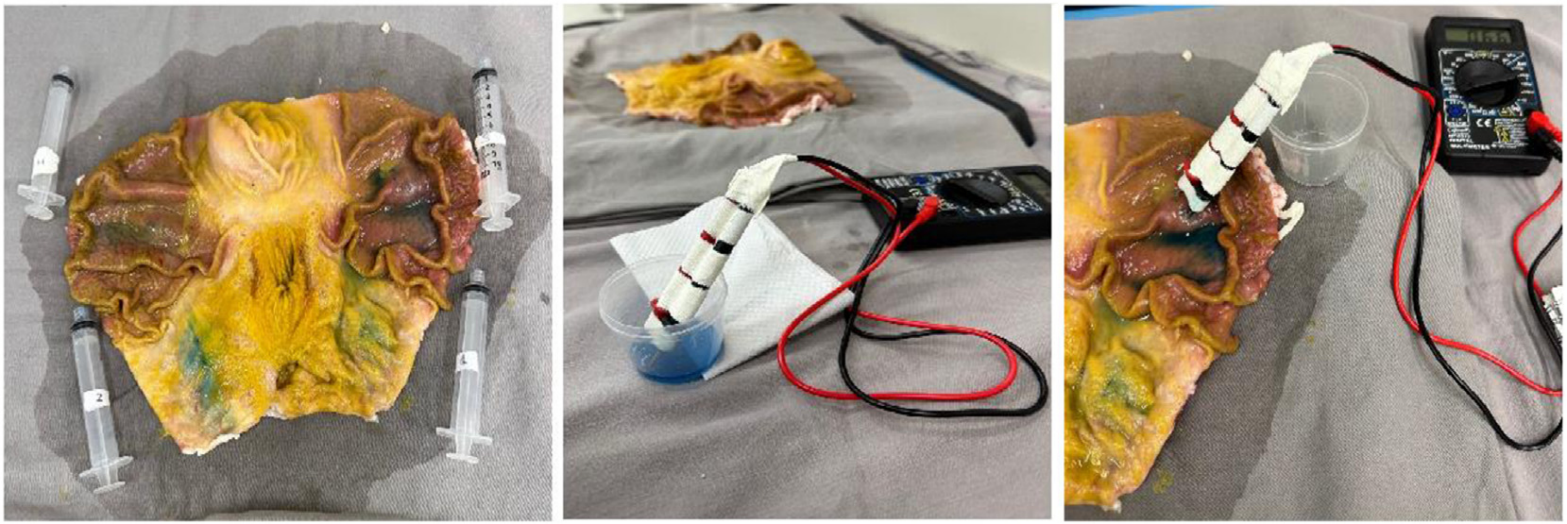
Pictures of measurement of the electrical resistance of pure solutions before and after injection into the submucosa of ex vivo gastric tissue. São Paulo, 2024.

In Phase 3, electrical conduction was assessed by measuring electrical resistance using a digital multimeter on the 2000K Ohms (Ω) scale, both in the solutions and after the injection of 5 mL into the submucosa of ex vivo gastric tissue (Fig. 1).

The first solution is Blue Eye (Blue Eye), which consists of a .4% hyaluronic acid mixed with methylene blue. Hyaluronic acid is a glycosaminoglycan found in connective tissue, known for its high viscosity and water retention capacity.^5^

The second option is a solution containing 6% hydroxyethyl starch typically used as an intravenous volume expander (known as Voluven, manufactured by Fresenius Kabi, Bad Homburg vor der Höhe, Germany). It is commonly used off-label for ESD.^1^

Based on our experience as a center in Brazil, mannitol is a suitable substance for endoscopic resection procedures because of its favorable osmotic properties and excellent retention.^8^ Sodium chloride solution is indeed widely used in clinical settings for its properties as an isotonic solution. It is important to note that although .9% is the most common concentration, it can also be used in other concentrations (eg, .45%). These different concentrations can have varying effects on physiological processes and may influence factors such as osmolarity and fluid balance.

The solutions tested were thus as follows: Blue Eye (B-bluee), Voluven (Voluv; 6% hydroxyethyl starch solution), 10% mannitol with .45% sodium chloride (2.5 mL of 20% mannitol solution + 2.5 ml of .9% NaCl [Ma-Na45]), and 20% mannitol with .9% sodium chloride (212 mL of 20% mannitol + 10 mL of 20% NaCl [Ma-Na90]).

The Voluv, Ma-Na45, and Ma-Na90 solutions were mixed with methylene blue dye to retain consistent color tones for all substances evaluated by examiners.

## Results

### Phase 1

**Figure 2:**
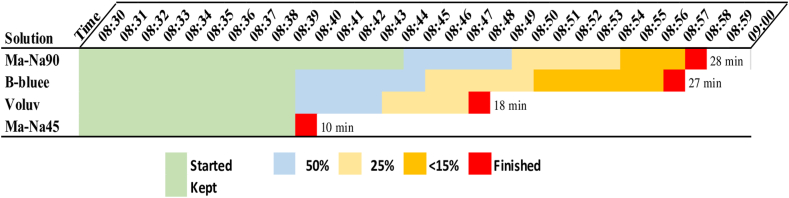
Phase 1 for the maintenance time of a cushion with 4 different endoscopic solutions to the porcine submucosa. São Paulo, 2024. *Ma-Na90*, 20% mannitol with .9% sodium chloride; *B-bluee*, Blue Eye; *Voluv*, Voluven; *Ma-Na45*, 10% mannitol with .45% sodium chloride.

Among the 4 evaluated solutions, Ma-Na90 displayed the longest durability with a time of 28 minutes. In second place, B-bluee displayed a duration of 27 minutes, followed by Voluv with a time of 18 minutes. Ma-Na45 exhibited the shortest duration, maintaining for only 10 minutes. Ma-Na45 had a rapid absorption, while the other solutions were gradually absorbed with a slow cushion reduction (Fig. 2).

### Phase 2

**Figure 3:**
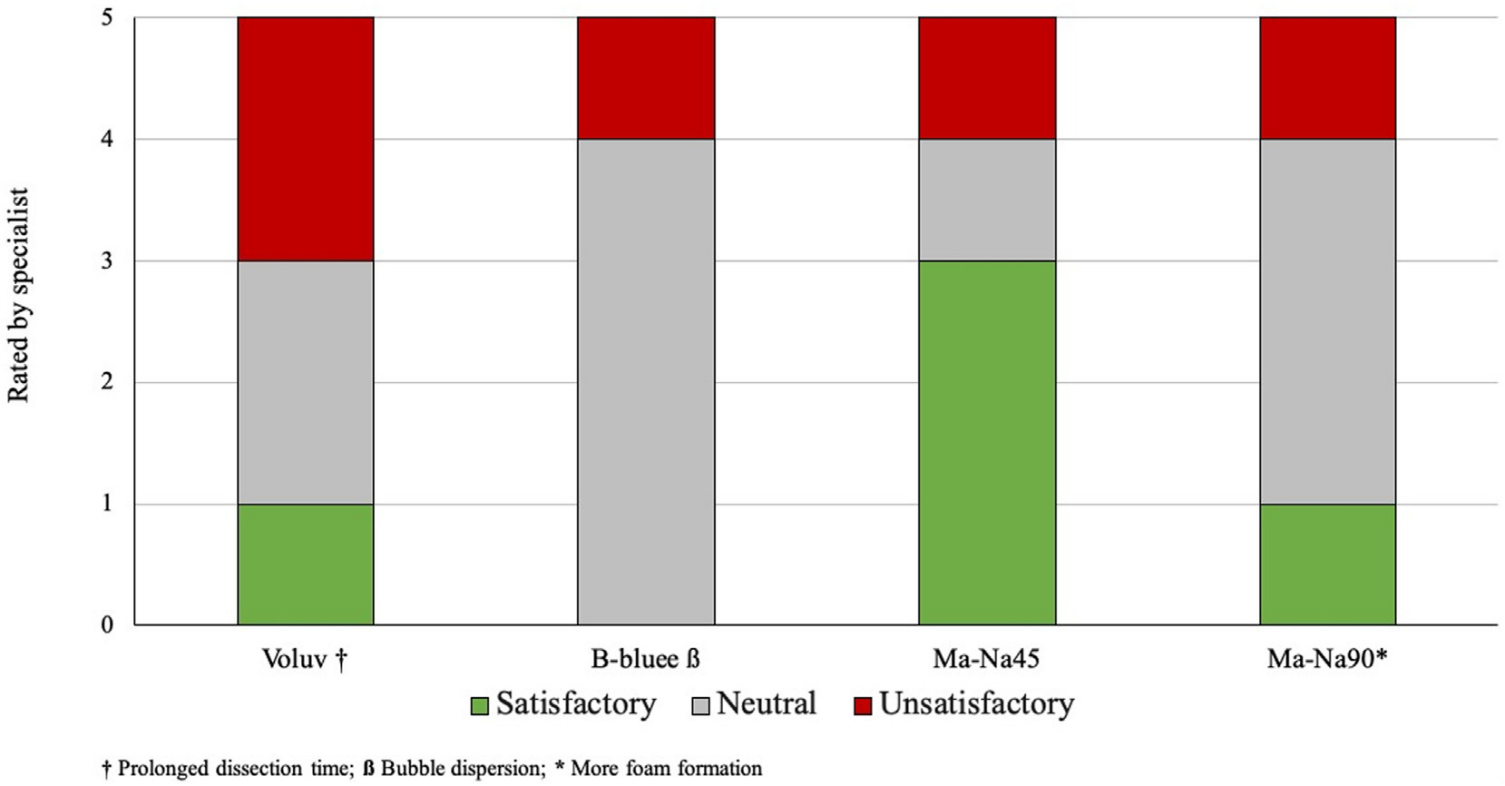
Bar chart of the endoscopists’ satisfaction with the solution quality after the procedure on the porcine submucosa (double blinded and randomized). São Paulo, 2024. *Voluv*, Voluven; *B-bluee*, Blue Eye; *Ma-Na45*, 10% mannitol with .45% sodium chloride; *Ma-Na90*, 20% mannitol with .9% sodium chloride.

In the second phase of the study, analysis of the results showed that Ma-Na45 was viewed as satisfactory, whereas Voluv was unsatisfactory. B-bluee was viewed as neutral according to the endoscopists' opinion after the experimental procedure (Fig. 3). Among the reasons for specialist dissatisfaction, our findings indicate that the short duration and rapid bubble dispersion were the primary explanations (Table 1). It was observed that Ma-Na90 generated a significantly greater amount of foam in 3 of the 5 random blinded tests (60%). Furthermore, Voluv required substantially more time for dissection; we observed that 60% of the prolonged dissections were on Voluv experiments. No perforations occurred during the procedures.

**Table 1:** Endoscopists’ assessment of the solution quality after the procedure on the porcine submucosa (double blinded and randomized); São Paulo, 2024

	Voluv	B-bluee	Ma-Na45	Ma-Na90
Prolonged dissection time	3 (60)	1 (20)	2 (40)	0 (0)
More foam formation	1 (20)	1 (20)	1 (20)	3 (60)
Bubble dispersion	1 (20)	3 (60)	2 (40)	2 (40)
Rated by specialist				
Satisfactory	1 (20)	0 (0)	3 (60)	1 (20)
Neutral	2 (40)	4 (80)	1 (20)	3 (60)
Unsatisfactory	2 (40)	1 (20)	1 (20)	1 (20)

Values are n (%).

*Voluv*, Voluven; *B-bluee*, Blue Eye; *Ma-Na45*, 10% mannitol with .45% sodium chloride; *Ma-Na90*, 20% mannitol with .9% sodium chloride.

### Phase 3

**Figure 4:**
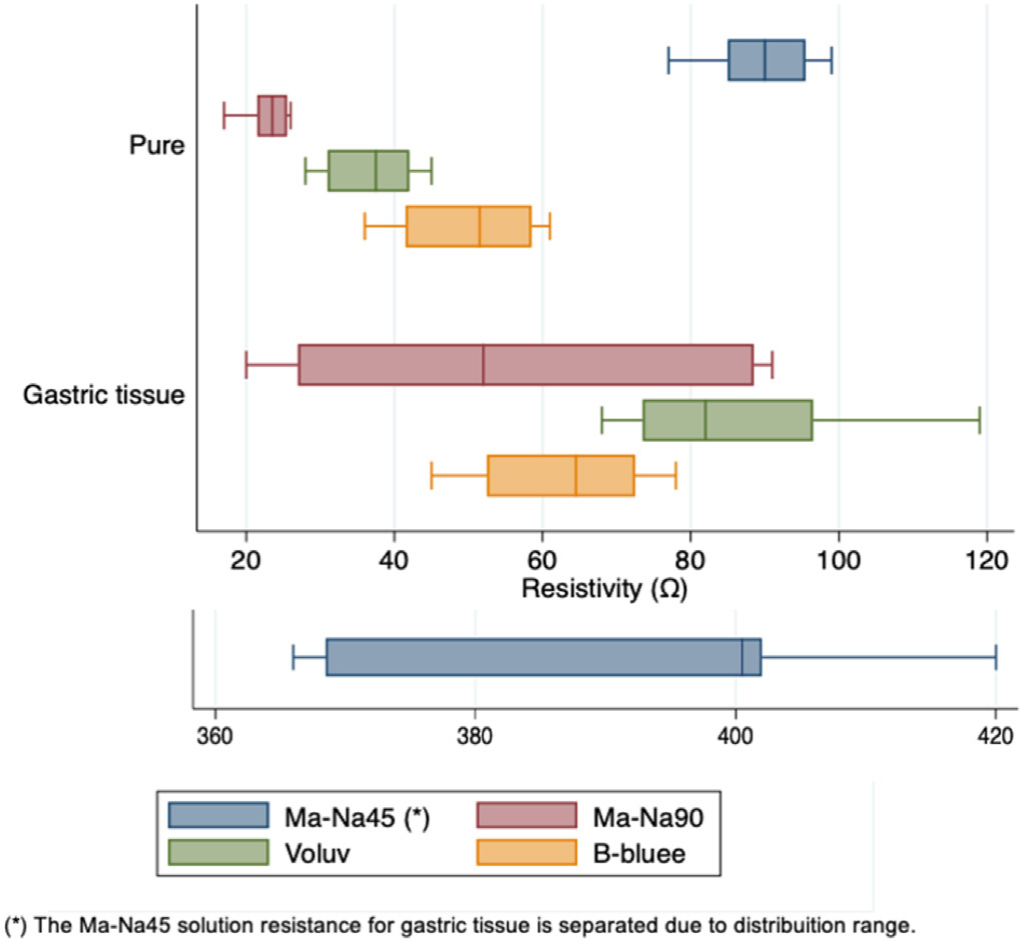
Boxplot with the distribution of the measurement of resistivity of 4 submucosal injection fluids of the gastric tissue compared with the pure endoscopic solutions. São Paulo, 2024. *Ma-Na45*, 10% Mannitol with .45% sodium chloride; *Ma-Na90*, 20% mannitol with .9% sodium chloride; *Voluv*, Voluven; *B-bluee*, Blue Eye.

In the third phase of the experiment, 8 measurements were taken for each pure solution and after injection into the piece of ex vivo porcine gastric tissue. The boxplot in Figure 4 presents the distribution data observed for the pure solution and the gastric tissue. Ma-Na45 resistivity on the tissue had an amplitude range between 366 and 420 Ω; it exhibited the highest average electrical resistance recording (89.62 Ω) on the pure solution, whereas in the tissue, it exhibited an average electrical resistance of 391 Ω (median, 400.5 Ω; interquartile range, 33.5 Ω). Ma-Na90 showed the lowest dispersion electrical resistance on the solution, with only 23 Ω, and in the corresponding tissue, it presented a bigger amplitude with an average of 55.75 Ω (median, 52 Ω; interquartile range, 61.5 Ω). The resistivity distribution of each solution is described in Table 2.

**Table 2:** Measurement of resistivity of 4 porcine submucosal injection fluids of the gastric tissue compared with the pure endoscopic solutions; São Paulo, 2024

	Ma-Na90 (n = 5)		B-bluee (n = 5)		Voluv (n = 5)		Ma-Na45 (n = 5)	
	Pure	Gastric tissue	Pure	Gastric tissue	Pure	Gastric tissue	Pure	Gastric tissue
Resistivity (Ω)								
Minimum	17	20	36	45	28	68	77	366
First quartile (25%)	21.5	27	41.5	52.5	31	73.5	85	368.5
Median (50%)	23.5	52	51.5	64.5	37.5	82	90	400.5
Third quartile (75%)	25.5	88.5	58.5	72.5	42	96.5	95.5	402
Maximum	26	91	61	78	45	119	99	420
Interquartile range (Q3-Q1)	4	61.5	17	20	11	23	10.5	33.5
Average	23	55.8	50	62.8	36.8	86.4	89.6	391
Standard deviation	3	30.6	9.7	12.02	6.3	17.5	7.3	20.4

*Ma-Na90*, 20% Mannitol with 0.9% sodium chloride; *B-bluee*, Blue Eye; *Voluv*, Voluven; *Ma-Na45*, 10% mannitol with 0.45% sodium chloride; *Q3-Q1*, quartile 1 to quartile 3.

## Discussion

The characteristics outlined in this experimental model are important for evaluating the best injectable solution. In phase 1, Ma-Na90 showed the highest durability in cushion formation while Ma-Na45 showed the lowest. Although Ma-Na45 had a shorter and more abrupt presentation time for the reduction of bubbles, we found that it was the one that best satisfied the experts in the randomized double-blind trial.

This emphasizes the importance of choosing a solution that provides a stable cushion for a sufficient duration to facilitate lesion resection and reduce the number of injections needed to maintain the elevated cushion. Although there is consistent evidence that hypertonic and viscous solutions increase the duration of the submucosal cushion, their benefits in relation to clinical outcomes are more controversial.^10^^,^^14^^,^^21^

In phase 2, Ma-Na45 was considered satisfactory, while Voluv was unsatisfactory. Ma-Na90 was more related to foam formation, an interesting fact as this is something that hinders visualization and adds more time for cleaning and technical feasibility, and thus has practical implications for conducting the procedure. Voluv was associated with the longest dissection time and is also a subjective parameter that involves skills with implications and limitations. In Phase 3, Ma-Na45 exhibited the highest average electrical resistance, indicating lower conductivity in current transmission.^11^

The electrical resistance of solutions is an influential parameter, especially considering the need for electrical current transmission during resections, which is often overlooked. Despite its recognition by many endoscopists, there is still a lack of data on the electrical properties of substances and the exact relationship between the electrical performance of submucosal injection.^15^^,^^22^ It is known there is a relationship between the principles of electrocautery energy and common adverse events observed in endoscopy, such as perforation, bleeding, and post-polypectomy syndrome.^11^^,^^23^^,^^24^ Beyond these variables, the selection of the optimal solution will hinge on various factors, including the endoscopist's preferences, substance availability, and the costs of the products.^3^^,^^11^^,^^14^

Several potential limitations could be considered when interpreting the current study. The study had constraints such as porcine models, a limited number of procedures, and subjective endoscopists' assessments; however, this study establishes a groundwork for selecting the best solution in endoscopic procedures. In phase 3, which used ex vivo porcine stomachs, the absence of factors such as blood flow, peristalsis, respirophasic tissue movement, or other physiological variables may have influenced how fluid diffused into the tissue and potentially affected resistivity. Additional comparative studies are essential to determine the optimal solution, considering that only 5 experiments were conducted for each solution in this analysis and only 2 endoscopists were involved.

This experiment illustrates the complexity of selecting the optimal solution based on a combination of variables. It is noteworthy that the use of Ma-Na90 stands out as a prominent option among the solutions due to its favorable overall results and easy accessibility.

## Ethics Statement

We declare that the Água Branca Group, located at Estrada Municipal Itu 216, S/N, Pedregulho, Itu-SP, holder of CNPJ 07.937.313/0001-10 and State Registration 387.187.160.118, registered with CRMV-SP No. 46886, works exclusively with animals and supplies, and is responsible for selecting animals (healthy animals) and selling them with the issuance of GTA (Animal Transportation Guide — MAPA) for INCOR — Division of Experimentation of HCFMUSP, in accordance with the request and in compliance with Animal Welfare conditions as per current legislation (Federal Law No. 11794). The responsibility for the collection and transportation of the animals lies with INCOR.

## Disclosure

All authors disclosed no financial relationships.
